# Glycan dependent phenotype differences of HIV-1 generated from macrophage versus CD4^+^ T helper cell populations

**DOI:** 10.3389/fimmu.2023.1107349

**Published:** 2023-06-21

**Authors:** Edwin J. Heeregrave, Jordan Thomas, Toni M. van Capel, Esther C. de Jong, Georgios Pollakis, William A. Paxton

**Affiliations:** ^1^ Laboratory of Experimental Virology, Department of Medical Microbiology, Amsterdam University Medical Center, University of Amsterdam, Amsterdam, Netherlands; ^2^ Department of Clinical Infection, Microbiology and Immunology, Institute of Veterinary and Ecological Sciences, University of Liverpool, Liverpool, United Kingdom; ^3^ Department of Experimental Immunology, University Medical Center, University of Amsterdam, Amsterdam, Netherlands

**Keywords:** HIV-1, macrophages, T helper, glycan, phenotypes

## Abstract

Human immunodeficiency virus type 1 (HIV-1) is able to infect a variety of cell types with differences in entry efficiency and replication kinetics determined by the host cell type or the viral phenotype. The phenotype of the virus produced from these various cell types, including infectivity, co-receptor usage and neutralisation sensitivity, may also be affected by the characteristics of the producing cell. This can be due to incorporation of variant cell-specific molecules or differences in post-translational modifications of the gp41/120 envelope. In this study we produced genetically identical virus strains from macrophages, CD4-enriched lymphocytes as well as Th1 and Th2 CD4^+^ cell lines and compared each different virus stock for their infectivity in various cell types and sensitivity to neutralisation. In order to study the effect of the producer host cell on the virus phenotype, virus stocks were normalised on infectivity and were sequenced to confirm env gene homogeneity. Virus production by Th1 or Th2 cells did not compromise infectivity of the variant cell types tested. We observed no difference in sensitivity to co-receptor blocking agents upon viral passage through Th1 and Th2 CD4^+^ cell lineages nor did this affect DC-SIGN-mediated viral capture as measured in a transfer assay to CD4^+^ lymphocytes. Virus produced by macrophages was comparably sensitive to CC-chemokine inhibition as was virus generated from the array of CD4^+^ lymphocytes. We identified that virus produced from macrophages was fourteen times more resistant to 2G12 neutralisation than virus produced from CD4^+^ lymphocytes. Macrophage-produced dual-tropic (R5/X4) virus was six times more efficiently transmitted to CD4^+^ cells than lymphocyte-derived HIV-1 (p<0.0001) after DCSIGN capture. These results provide further insights to what extent the host cell influences viral phenotype and thereby various aspects of HIV-1 pathogenesis but suggest that viruses generated from Th1 versus Th2 cells are consistent in phenotype.

## Introduction

Human immunodeficiency virus type 1 (HIV-1) differentially infects a variety of cell types, which can be partly explained by differences in CC- or CXC-chemokine receptor expression levels or CC-chemokine production (1–4). Passage through different cell lineages can affect virus infectivity, co-receptor usage and neutralisation sensitivity (5–9). This can be caused by incorporation of host cell-specific molecules or other differences in the viral envelope, partly caused by a differential production process (10–14). Many host cell proteins are incorporated into virions that can influence virus phenotype (11, 14). For example, HLA-DR incorporation increases particle infectivity and can also result in anergy and T cell apoptosis (15, 16). Additionally, incorporation of the gut-homing integrin α4β7 may play a key role in pathogenesis and transmission whilst also providing a potential target for novel therapies (17, 18). Similarly, differential glycosylation can also influence virus infectivity, transmission and neutralisation sensitivity (19–22).

Many studies have compared virus production by the monocyte/macrophage lineage versus lymphocytes. Monocytes have been shown to be less susceptible to HIV-1 than lymphocytes and have a lower daily virus production, but maintain virus production for longer due to a lower sensitivity to virus-induced apoptosis (23–26). Previous research has demonstrated that macrophage-derived HIV-1 strains bind to a different region of the CCR5 co-receptor than T cell-derived strains (27, 28). While lymphocyte-produced virus preferentially infects the autologous cell-type, monocyte/macrophage-produced virus equally infects monocytes/macrophages as well as lymphocytes (29). The observations in most of the preceding studies can be an effect of intra-patient viral evolution as well as differences in viral phenotypes attributable to cell-type of production. Few studies have used genetically homogenous viruses produced from different cells for their experiments. One of these studies found that macrophage-derived simian immunodeficiency virus (SIV) is more infectious than T cell-derived virus (30). Further, macrophage-produced virus was shown to possess a different glycosylation profile than T cell-derived virus, which can influence both infectivity and neutralisation sensitivity (30, 31). Macrophage-produced HIV-1 envelopes can contain a higher degree of carbohydrates as well as demonstrate a difference in the types of oligosaccharides present due to alterations in post-translation modifications between the cell types (31). Furthermore, macrophages generate virions that specifically incorporate CD36 as opposed to CD26 when produced by lymphocytes (11, 32, 33). This distinction was used to assess increased virus production by macrophages upon infection with *M. tuberculosis.*

Multiple groups have demonstrated that HIV-1 induces a switch from T helper 1 (Th1) to a Th2 or Th0 response, but such a switch has not been confirmed by studies from other groups (34–40). Th2 cells are preferentially infected by CXCR4 using virus and Th1 cells by CCR5 using variants, which correlates with chemokine receptor expression levels on these cell subsets (41, 42). Most but not all studies designate Th2 cells as better virus producers than Th1 cells (1, 43–45). Although Th1 cells express more CCR5 on their cell surface than Th2 cells, reduced replication in Th1 cells likely correlates with higher CC-chemokine levels in this cell type as well as increased expression of viral restriction factors such as APOBEC3G, TRIM22, TRIM5 and PPARγ (4, 46–48). The difference in gene expression profile between Th1 and Th2 cells demonstrates that these cell types differ in many characteristics, which can influence virus phenotype (49, 50).

Overall, there is limited knowledge regarding the specific contribution of the producer cell in modulating the phenotypic characteristics of HIV-1. In this study we aim determine the influence of macrophages, lymphocytes, Th1 and Th2 cells on the phenotype of the produced virus. To this end we infected these different cell types with identical virus strains and harvested virus at the peak of replication. These virus stocks were normalized on tissue culture infectious dose (TCID_50_) to correct for differences in infectivity prior to use in various assays. Additionally, genetically identical virus stocks were used to inoculate different producer cell types. As such, through this analysis, we aimed to determine the effect of the producer cell on virus glycosylation and consequently, infection phenotype, independent of virus genetic variation. We demonstrate that virus production by either T helper cell population did not influence infectivity for the other cell subset. Furthermore, virus produced by macrophages and lymphocytes possessed similar sensitivity to agents blocking the HIV-1 co-receptors. Transmission via dendritic cell-specific intercellular adhesion molecule 3-grabbing non-integrin (DC-SIGN) was enhanced for a dual-tropic macrophage-produced virus and sensitivity to 2G12 neutralisation was also affected by macrophage passage. These results provide us with more insight into the role of HIV-1 producer cells on viral phenotype.

## Materials and methods

### Generation of monocyte-derived macrophages and CD4-enriched lymphocytes

CD4 enriched lymphocytes and monocyte derived macrophages were isolated from peripheral blood mononuclear cells (PBMCs). PBMCs were isolated from a buffycoat of a single blood donor by Ficoll-Hypaque density gradient centrifugation. Cells destined to become macrophages were left to adhere for five days in RPMI 1640 medium supplemented with 10% human serum AB+, 20% fetal calf serum (FCS) and penicillin/streptomycin. To enrich for CD4 lymphocytes, PBMCs were cultured at 37°C in six well plates at a concentration of 2x10^6^ cells/ml. After five days, non-adherent cells were removed with three washes and the adherent cells were propagated for an additional two days to reach high confluence. PBMC were cultured in RPMI 1640 medium supplemented with 10% FCS and penicillin/streptomycin in addition of 100 units/ml interleukin 2 (IL-2). The cells were then phytohaemagglutinin (PHA)-activated (2 μg/ml) for three days, after which CD8^+^ lymphocytes were depleted using anti-CD8 immunomagnetic beads (Dynal, Invitrogen, Breda, The Netherlands). Cells were propagated at a concentration of 2x10^6^ cells/ml.

### Generation of Th1 and Th2 cells

PBMC were isolated from blood from a single blood donor by Ficoll-Hypaque density gradient centrifugation. Naïve CD4^+^ lymphocytes were isolated using the CD4^+^ T cell isolation kit containing a cocktail of biotin-conjugated monoclonal antibodies against CD8, CD14, CD16, CD19, CD36, CD56, CD123 and TCRγδ (Miltenyi Biotec B.V., Utrecht, The Netherlands). Non-CD4^+^ T cells were removed with anti-biotin microbeads and α-CD45RO was used to remove memory T cells with α-PE beads (DAKO, Heverlee, Belgium). Cell depletions were performed on the AutoMACS (Miltenyi). Naïve CD4^+^ T cells (2.5x10^5^ cells/ml) were stimulated with immobilized α-CD3 (1 μg/ml; CLB-T3/2 16A9) and α-CD28 (2 μg/ml; CLB-CD28/1 15E8; both from Sanquin, Amsterdam, the Netherlands) for ten days in the presence of cytokines. Cells were cultured in IMDM with 5% human serum, gentamycin and 10 units/ml IL-2. To generate Th1 cells, rIL-12 (0.5 ng/ml; R&D systems, Minneapolis, MN, USA) and a neutralizing antibody against IL-4 (1 μg/ml; 5B5, U-CyTech Biosciences, Utrecht, The Netherlands) were added to the culture while for Th2 cell generation rIL-4 (128 ng/ml; Biosource, Nivelles, Belgium) and an antibody against IL-12 (10 μg/ml; U-CyTech Biosciences, Utrecht, The Netherlands) were added. To generate fully polarized Th2 cells, a re-stimulation was performed for an additional ten days with PHA (2 μg/ml) and irradiated feeder cells in the presence of the same cytokines and antibodies. After a second round of polarization, cells were re-stimulated with PHA and irradiated feeder cells two days prior to HIV-1 infection or stored at -150°C for future experiments. Phenotype of the Th1 and Th2 cells was analyzed by flow cytometry.

### Virus stocks

PBMC-derived HIV-1 stocks (previously generated) were used to infect macrophages, CD4-enriched lymphocytes as well as Th1 and Th2 cells. We used stocks of CCR5 using SF162 and NSI-18, dual-tropic H671-B10 (51) and CXCR4 using LAI. Cells were infected with these virus strains with an end concentration ranging between 10^3^ and 10^4^ TCID_50_/ml, varying per strain. We initiated four or five parallel cultures derived from each cell type. Virus production was monitored daily by CA-p24 ELISA. At the peak of viral replication, virus was harvested, membrane filtered (0.2 μm) and aliquoted. We determined the TCID_50_ of each virus stock on CD4-enriched lymphocytes and further infections were normalized on TCID_50_ values (**Table 1**). The gp120 *env* gene of all virus stocks was sequenced using primers spanning the C2C4 region: 5’-GAAAGAGCAGAAGACAGTGGCAATGA-3’ and 3’-GTGCTTCCTGCTGCTCCTAAGA-5’. Population sequencing was performed by the Sanger method that does not detect all minor species, however it does have a sensitivity in doing so for minor species of 10% and higher and where the method is routinely used to detect drug resistant minor populations. In all virus stocks and post infection controls we did not detect any genetic shift. Therefore, we have concluded that differences in infectivity or virus phenotype can only result from post translational processing of the sugar moieties of the glycan shield.

### HIV-1 infection assay

All infections were performed in duplicate or triplicate in 96 wells format and input was normalized on TCID_50_. A non-replicative culture was included to correct for background CA-p24 values, which were determined approximately twice a week. Single-round TZM-bl (NIH AIDS Reagent and Reference Reagent Program) infections with luciferase read-out were performed to confirm equal infectivity of the TCID_50_ normalized virus stocks. Infections were conducted as previously described (3). Briefly, one day prior to infection, 2x10^4^ TZM-bl cells were plated in DMEM containing 10% fetal bovine serum, 1x minimum essential medium nonessential amino acids and penicillin-streptomycin (both at 100 units/ml). Virus (10^3^ TCID_50_) was added to the cells in the presence of 400 nM saquinavir (Roche, Mannheim, Germany) and 40 µg/ml DEAE, in a total volume of 200 µl. Two days post-infection, the cells were washed, lysed and luciferase activity was measured using a luciferase assay kit (Promega, Madison, WI, USA) and a Glomax luminometer according to the manufacturer’s instructions (Turner BioSystems, Sunnyvale, CA, USA). Uninfected cells were used to correct for background luciferase activity.

### Virus inhibition and neutralisation

Chemokine receptor blocking experiments and antibody neutralisation experiments were performed in quadruplicate and in 96 wells format. Chemokine receptor blocking experiments were performed using RANTES (regulated on activation normal T cell expressed and secreted; Biosource, Nivelles, Belgium) and AMD3100, a CXCR4 antagonist (kind gift from D. Schols). CD4-enriched lymphocytes (2.5x10^5^ cells) were incubated for 30’ at 37°C with 2-fold dilutions of the respective chemokine. Virus was added at a concentration of 400 TCID_50_. At days 4, 7, 10 and 14, virus replication was measured using CA-p24 ELISA in the cultures without chemokines. At the peak of viral replication, CA-p24 values of all chemokine dilutions were determined and inhibition curves were constructed with automatic outlier elimination. The 50% and 90% inhibitory concentrations (IC_50_ and IC_90_, respectively) were determined using version 5.01 of GraphPad Prism software (San Diego, CA, USA). 2G12 (Polymun SIF, Vienna, Austria) neutralisations were performed in the same manner but virus (400 TCID_50_) was first incubated with 2-fold antibody dilutions for 30’ at 37°C, after which cells were added (2.5x10^5^ cells). Statistical significance was calculated using the Mann-Whitney U test and p-values smaller than 0.05 were regarded as significant.

### HIV-1 *trans*-infection as a measure of viral capture via DC-SIGN

In order to study the ability of different viruses generated in variant cell lineages to interact with DC-SIGN we utilised an *in vitro* model of cell capture and subsequent transfer to CD4^+^ T lymphocytes, even though this mechanism may not occur *in vivo*. Transmission experiments were performed in triplicate using the DC-SIGN expressing Raji cell line (Raji-DC-SIGN) with Raji cells as negative controls (kind gift from T. Geijtenbeek). These cells were propagated in RPMI 1640 medium supplemented with 10% FCS and penicillin/streptomycin. DC-SIGN expression was induced with neomycin (2 mg/ml) and routinely monitored using flow cytometry. Virus (10^3^ TCID_50_ end concentration) was incubated with Raji-DC-SIGN cells for two hours at 37°C, after which unbound virus was removed by washing. Approximately 9x10^4^ Raji cells were subsequently applied to 2x10^5^ CD4-enriched lymphocytes to allow viral transmission. Cells were cultured in RPMI 1640 supplemented with 10% FCS and IL-2 (100 units/ml) in addition of penicillin/streptomycin. After two days, medium was refreshed and indinavir (1μM) was added to facilitate virus detection, through preventing viral re-infection but not accumulation of intracellular p24 used for monitoring infection levels. After four days of transmission, cells were prepared for flow cytometry analysis.

### FACS analysis

Cells were washed and fixed with 3.7% paraformaldehyde for 20 min after which the fixative was quenched with 20 mM glycine. Cells analyzed for intracellular cytokine analysis were first treated for 6 hours with PMA (10 ng/ml) and ionomycin (1 μg/ml; both Sigma-Aldrich, Zwijndrecht, the Netherlands) prior to fixation. Brefeldin A (10 μg/ml; Sigma-Aldrich) was added for 4.5 hours. Cells were permeabilized in PBS containing 0.1% saponin, 1% bovine serum albumin and 50mM NH_4_Cl and subsequently stained with allophycocyanin (APC)-labelled α-CD3 (BD Biosciences, Breda, The Netherlands) and FITC-labelled α-CA-p24 (Beckman Coulter, Fullerton, CA, USA). Excess antibody was washed away and 1.5x10^5^ cells were analyzed by flow cytometry. Statistical significance was calculated using the two-tailed Wilcoxon signed rank test and values smaller than 0.05 were regarded as significant.

## Results

### Harvest generation of virus stocks and infectivity

**Figure 1:**
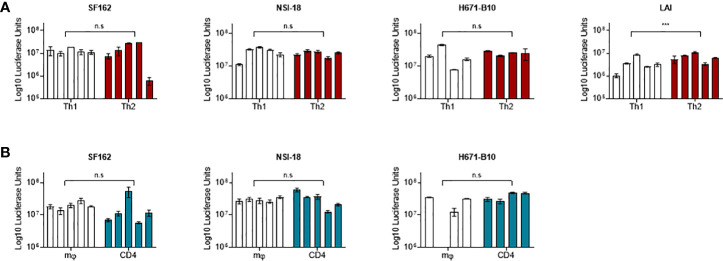
TZM-bl cell infections. TZM-bl cell infections using 1,000 TCID_50_ virus clones were measured by luciferase activity, depicted in log scale on the y-axis. **(A)** Th1-produced (white) and Th2-produced (red) virus (n=5). **(B)** Macrophage-produced (mφ) (white) and lymphocyte-produced (CD4) (blue) virus (n=5). Virus clones were produced in five replicates for each producing cell type and were used to infect TZM-bl cells in triplicate. For each virus isolate replicate, the median value of a triplicate infection is shown as a single bar on the graph with error bars representing the range. ***, P<0.001; ns, not significant.

To study the influence of producer cell type on the derived HIV-1 phenotype we generated numerous virus stocks (representing R5, R5X4 and X4 variants) on macrophages, CD4-enriched lymphocytes and Th1 as well as Th2 CD4^+^ lymphocytes (**Table 1**). Viruses were harvested typically two weeks after infection. To rule out the possibility that sequence differences influenced our findings, we sequenced the gp120 env gene of ten virus stocks and found no evidence of viral evolution when compared to input virus sequence. HIV-1 infection did not affect the cytokine profile of the T helper CD4^+^ cells, since flow cytometry analysis two days after virus infection confirmed that the cells preserved their polarized cytokine expression profile (data not shown). The harvested viruses were normalized on TCID_50_ and assessed for infectivity on TZM-bl cells. Cells were infected with each virus stock and we paired the infectivity between macrophage-produced versus lymphocyte-produced virus and also between Th1- and Th2-produced stocks (**Figure 1**). Normalization on TCID_50_ resulted in comparable infectivity of the virus stocks produced by different cell types with the exception of Th1- and Th2-produced LAI. We observed a statistically significantly higher infectivity of Th2-produced variants over Th1-produced viruses. Despite this statistical significance, the difference between both stocks is small and the infectivity of three variants from each cell type was similar.

**Table 1:** Virus stocks.

Virus	Macrophage		CD4		Th1		Th2	
	TCID_50_ ^a^	CA-p24^b^	TCID_50_	CA-p24	TCID_50_	CA-p24	TCID_50_	CA-p24
**SF162_1**	31,550	27	104,472	42.8	4,159	4.1	31,550	8.9
**SF162_2**	14,093	22.2	**104,472**	94	9,311	3.3	**20,845**	12.7
**SF162_3**	20,845^c^	25.6	20,845	53.5	830	1.3	14,093	15.9
**SF162_4**	**20,845**	18.9	104,472	52.8	**1,858**	2.8	6,295	9.1
**SF162_5**	14,093	20.3	70,632	42.8	1,256	3.5	154,525	12.5
**NSI-18_1**	2,812	13.3	20,845	46.5	31,550	9.4	46,666	12.8
**NSI-18_2**	6,295	27	46,666	60	9,311	12.2	31,550	25.7
**NSI-18_3**	**6,295**	17.7	**46,666**	53.8	**14,093**	14.3	**31,550**	24.2
**NSI-18_4**	14,093	17.3	104,472	47	14,093	13.8	46,666	17.4
**NSI-18_5**	4,159	17.9	70,632	52.3	20,845	9.9	31,550	14.2
**H671-B10_1**	**4,159**	5.6	6,295	4.8	**6,295**	13.1	**1,858**	11.6
**H671-B10_2**	**2,812**	3.2	**6,295**	3.2	4,159	15.4	2,812	10.1
**H671-B10_3**	1,858	3.2	**4,159**	4.2	20,845	13.3	1,858	13.5
**H671-B10_4**	9,311	4.9	4,159	3.8	**9,311**	11.9	**2,812**	10.9
**LAI_1**	n.p.^d^	n.p.	n.p.	n.p.	523,600	78	154,525	173
**LAI_2**	n.p.	n.p.	n.p.	n.p.	**154,525**	39.1	**154,525**	99
**LAI_3**	n.p.	n.p.	n.p.	n.p.	70,632	43.6	104,472	107
**LAI_4**	n.p.	n.p.	n.p.	n.p.	233,884	38.1	233,884	156
**LAI_5**	n.p.	n.p.	n.p.	n.p.	233,884	40.6	154,525	204

a TCID5 0 is given per ml and determined at day 7.

b CA-p24 in ng/ml.

c median values in bold. For H671-B10: the average of the middle two values were calculated.

d n.p. not performed.

### Th1- and Th2-derived HIV-1 isolates are equally infectious for the alternate T helper cell type

**Figure 2:**
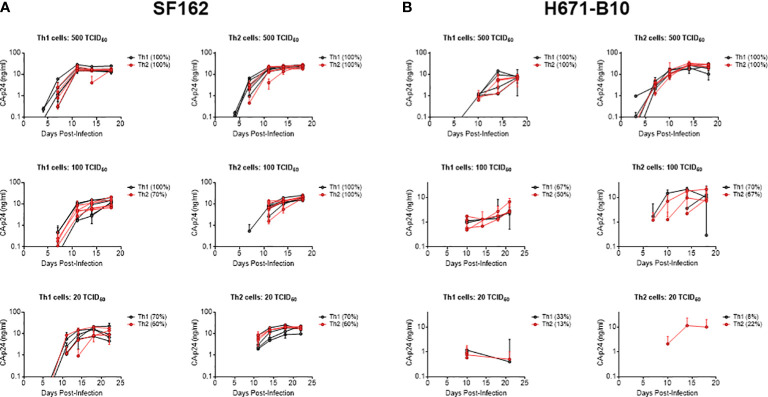
Infection of T helper cells with Th1- and Th2-produced HIV-1. SF162 and H671-B10 virus clones were produced in four or five replicates from Th1 and Th2 cells and used to infect either Th1 or Th2 cells in duplicate. Each line represents a virus clone replicate that established a productive infection, with Th1-produced virus presented in black and Th2-produced virus in red. The percentage of isolates resulting in productive infection is displayed in brackets. Infections were performed with three different TCID_50_ values; 500, 100 and 20, of SF162 (**A**, CCR5 using) and H671-B10 (**B**, dual-tropic). CA-p24 production is depicted on the y-axis in logarithmic scale over the course of infection.

Differences in inclusion of cell-specific molecules into HIV-1 particles has been shown to modulate virus phenotypes from monocyte/macrophage-derived versus lymphocyte-derived virus (14, 30). To compare the infectivity of virus produced by Th1 and Th2 cells, we infected both T helper populations with virus produced by these cell types. We included CCR5 using SF162 and dual-tropic H671-B10. We performed infections using four or five separate virus stocks from each cell type. For both viruses, all virus stocks replicated in a very similar manner on either cell type and reached comparable CA-p24 end values (**Figures 2A, B**). The percentage of variants that established productive infection also did not differ (**Figure 2**). Viruses produced by one T helper population did not preferentially replicate on the autologous cell type, although higher dilutions of H671-B10 seemed to have a minor preference for replication on cells they were produced by.

### HIV-1 produced by macrophages and lymphocytes possess comparable sensitivity to co-receptor blocking agents

**Figure 3:**
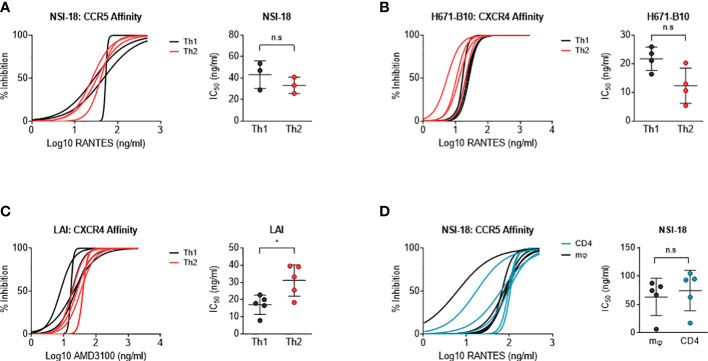
Determination of co-receptor affinity. Affinity for both the CCR5 and CXCR4 co-receptor was determined by HIV-1 infection of CD4-enriched lymphocytes in addition of 3-fold dilutions of RANTES and AMD3100, respectively, up to fully blocking concentrations. Logarithmic values are presented on the x-axis, while the y-axis depicts the percentage of inhibition. In a separate graph, we compared IC_50_ values between both viral stocks using the Wilcoxon signed rank test. Virus clones were produced in each cell type in 3, 4 or 5 replicates and used to infect each cell type in inhibition assays in quadruplicate. Each virus clone replicate is plotted as a single line and the IC_50_ value derived from these inhibition curves are plotted as a single point for each virus isolate replicate. **(A)** RANTES inhibition of Th1- (black) and Th2-produced (red) virus stocks (n=3) of NSI-18. **(B, C)** AMD3100 inhibition of Th1- (black) and Th2-produced (red) viral stocks of H671-B10 (dual-tropic) (n=4) and LAI (CXCR4 using) (n=5). **(D)** RANTES inhibition of macrophage (mφ)- (black) and lymphocyte-produced (blue) viral stocks of NSI-18 (CCR5 using) (n=5). *, P<0.05; ns, not significant.

We next investigated whether virus produced by different cell types influenced co-receptor usage. CD4-enriched lymphocytes were infected with Th1- and Th2-produced virus as well as macrophage- and lymphocyte-produced HIV-1, in the presence of increasing concentrations of blocking agents. RANTES was used to block the CCR5 co-receptor and AMD3100 was added as a CXCR4 antagonist. Th1- and Th2-produced NSI-18 demonstrated comparable sensitivity to RANTES, which was confirmed by inhibitions with SF162 (**Figure 3A** and data not shown). Opposing trends for CXCR4 usage were observed for dual-tropic H671-B10 and CXCR4 using LAI (**Figures 3B, C**). While Th1-produced H671-B10 had a 1.9-fold higher affinity for CXCR4 than Th2-produced virus, Th1-produced LAI had a 1.9-fold lower affinity than Th2-produced virus (P=0.0317) (**Figures 3B, C**). We observed similar inhibition values for LAI when we repeated the AMD3100 inhibition experiment. No difference in CCR5 affinity was observed between macrophage and CD4-derived variants (**Figure 3D**). IC_90_ estimates (data not shown) confirmed our observations that were based on IC_50_ calculations, concluding that virus passage through different cell types was barely influencing sensitivity to chemokine receptor blocking agents.

### Similar DC-SIGN-mediated transmission to T cells of Th1- and Th2-produced HIV-1, while macrophage-derived dual-tropic virus is preferentially transmitted over lymphocyte-derived virus

**Figure 4:**
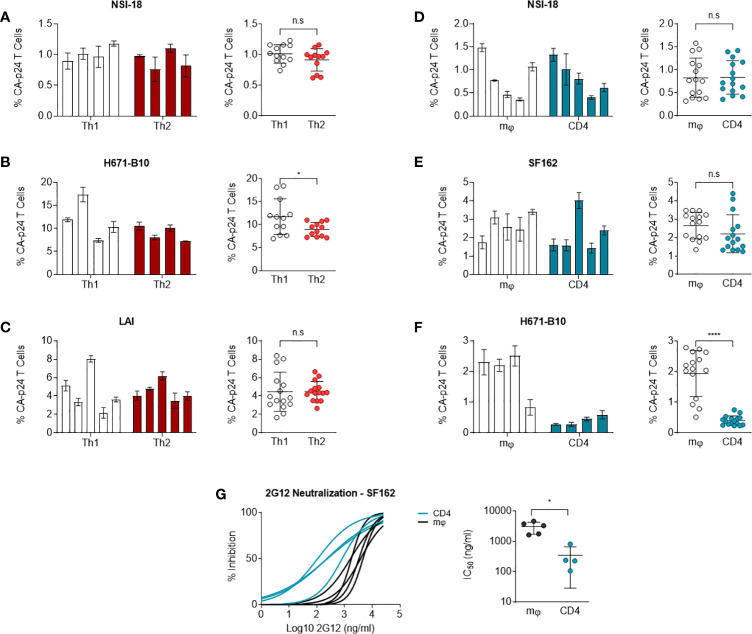
DC-SIGN-mediated transmission to CD4-enriched lymphocytes. NSI-18, H671-B10 and **(A–C)** Transmission of Th1- (white) versus Th2-produced (red) NSI-18 (CCR5 using) (n=4), H671-B10 (dual-tropic) (n=4) and LAI (CXCR4 using) (n=5). Three to five clones were produced from each cell type and infection experiments were performed in triplicate. The bars represent median values of HIV-infected lymphocytes for each clone. A separate graph depicts the values of all clones from each cell type and we used the Wilcoxon signed rank test to determine statistical significance on transmission of Th1- and Th2-produced variants. **(D–F)** Transmission of macrophage (mφ)- (white) versus lymphocyte-derived (blue) NSI-18 (CCR5 using) (n=5), SF162 (CCR5 using) (n=5) and H671-B10 (dual-tropic) (n=4). Transmissions with NSI-18 and H671-B10 were repeated once. The bars represent median values of HIV-infected lymphocytes for each clone. A separate graph depicts the values of all clones from each cell type and we used the Wilcoxon signed rank test to determine statistical significance on transmission of mφ - and lymphocyte-produced variants. **(G)** Sensitivity of macrophage (mφ)- (white) and lymphocyte-derived (blue) SF162 HIV-1 to the carbohydrate dependent 2G12 antibody neutralisation was determined by infecting CD4-enriched lymphocytes with virus, which was neutralized with 3-fold increasing concentrations of antibody. Inhibition curves were constructed based on CA-p24 values from the peak of viral replication. The experiment was conducted twice with one representative profile shown. *, P<0.05; ****, P<0.0001; ns, not significant.

Carbohydrates on HIV-1 envelope gp120 bind DC-SIGN, a C-type lectin, which can enable for virus binding to Raji cells expressing DC-SIGN and transmit HIV-1 to CD4^+^ T cells (52). This assay can be utilised as a means of monitoring the capacity of DC-SIGN to interact with virus or more likely virus Env antigen. Since virus produced by different cell types can result in variant degrees of glycosylation or post-translational modifications (31), we tested whether our produced isolates were transmitted by DC-SIGN with different efficiency. We incubated DC-SIGN expressing cells with HIV-1 and co-cultured these cells with CD4-enriched lymphocytes to monitor virus transmission. Using flow cytometry, we quantified HIV-1 infection levels of lymphocytes and we calculated the percentage of CA-p24 positive cells. Viruses produced by Th1 and Th2 cells were transmitted equally to CD4-enriched lymphocytes, apart from 671-B10 which showed a difference between Th1 and Th2 produced virus but most likely due to a higher transfer of two replicates (**Figures 4A–C**). Surprisingly, Th1-produced LAI demonstrated high variation in transmission by the different produced stocks, with up to a 4-fold difference. This occurred despite normalization on TCID_50_ and all stocks showed a comparable pattern of replication and were harvested at the same day with similar CA-p24 values. We also performed transmission experiments using macrophage- and lymphocyte-derived viral stocks that were either CCR5 using or dual-tropic. No significant difference in transmission of CCR5 using virus was observed (**Figures 4D, E**). Macrophage-produced dual-tropic variant H671-B10 however, was preferentially transmitted over lymphocyte-produced virus (p<0.0001; **Figure 4F**). Viral replication, day of harvest CA-p24 values and TCID_50_ of both viral stocks were comparable, indicating a true difference in transmission of this virus between macrophages and lymphocytes. We therefore conclude that CCR5 using HIV-1, produced by macrophages and lymphocytes, are equally transmitted to lymphocytes via DC-SIGN, while macrophage-produced dual-tropic virus is preferentially transmitted over lymphocyte-produced virus.

### Macrophage-produced HIV-1 is more resistant to 2G12 inhibition

To determine whether macrophage-produced and lymphocyte-produced viruses differ in sensitivity to antibody neutralisation, we conducted neutralisation experiments with 2G12, a carbohydrate-binding antibody. Differences in HIV-1 gp120 envelope glycosylation patterns can influence the sensitivity of virus to antibody neutralisation with 2G12 (53–58). We incubated our CD4 lymphocyte or macrophage generated SF162 virus stocks with 3-fold dilutions of 2G12 up to fully blocking concentrations and subsequently infected CD4-enriched lymphocytes. Macrophage-produced SF162 HIV-1 was 14-fold more resistant to neutralisation than virus produced by lymphocytes (P= 0.0159) (**Figure 4G**). We repeated the experiment with the same virus stocks and again observed that macrophage-derived virus was more resistant to 2G12 neutralisation (one representative profile shown). This result indicates that producer cell type can influence the sensitivity of HIV-1 to antibody neutralisation in a virus phenotype restricted manner.

## Discussion

In this study, we have analyzed the influence of the HIV-1 producer cell on virus phenotype. Virus production by Th1 or Th2 cells did not compromise infectivity for the alternate cell subset and these virus stocks were comparably sensitive to co-receptor blocking agents. We also observed similar levels of DC-SIGN-mediated transmission for viruses produced in both Th1 and Th2 cells. Virus produced by macrophages was comparable in sensitivity to CC-chemokine inhibition as lymphocyte-derived virus, but was 14x more resistant to 2G12. Macrophage-produced dual-tropic virus demonstrated 6x enhanced transmission via DC-SIGN than lymphocyte-derived HIV-1 (p<0.0001), but no significant difference was observed with CCR5 using variants.

Previously described differences in co-receptor usage patterns of HIV-1 derived from macrophages as opposed to lymphocytes is in all likelihood due to viral evolution within these patients and not solely an effect of the producer cell. In previous studies, virus has often been isolated from an anatomically occluded tissue such as the brain. Brain-derived variants differ in *env* gp120 sequence from lymphocyte-derived HIV-1, explaining the differences in virus phenotype (6, 59). In our study, CXCR4 usage of Th1-produced LAI is comparable with that of dual-tropic H671-B10, with IC_50_ values approaching 20 ng/ml. It is unclear why Th2-produced LAI has a 3-fold higher IC_50_ than Th2-produced H671-B10. H671-B10 can also infect cells via CCR5, so entry of Th2 cells via this co-receptor partly compromises entry via CXCR4 and therefore, reduced entry using CXCR4 may affect the sensitivity to AMD3100 inhibition. Such an effect was not observed with Th1 cells. Perhaps higher levels of CC-chemokines induces H671-B10 to predominantly enter Th1 cells via CXCR4, which may then result in equal IC_50_ values of H671-B10 and LAI.

The wide variation in transmission via DC-SIGN among Th1-produced variants of LAI, but also in other cell cultures, could indicate that differences in glycosylation exist among parallel infected cells. Virus stocks were normalized on TCID_50_ to exclude the influence of differences in infectivity. The disparity in outcome of DC-SIGN-mediated transmission between CCR5 using strains and the dual-tropic H671-B10 strain points to involvement of a viral factor. If it were only a host cell effect we would also have observed differences in transmission between CCR5 using viruses. Whether this phenomenon is specific for dual-tropic viruses remains to be determined. Since gp120 envelope proteins of variant HIV-1 strains can differ in their N-linked glycosylation profile (60), differences in glycosylation activity amongst cell types may affect some virus strains more than others. Apparently, a specific characteristic of the H671-B10 strain resulted in this difference, possibly being CXCR4-mediated signalling. Furthermore, we propose to treat the virus stocks with an endo-H glycosidase to determine the presence of mannose residues, which could help explain for the differential transmission observed between the viruses. Transmission of all macrophage-produced viruses resulted in 10-20% higher CA-p24 intensity over lymphocyte-produced virus, which indicates a replication advantage to these variants. Some Th1-produced virus stocks also demonstrated a 10% higher CA-p24 intensity in infected lymphocytes. It is tempting to speculate that macrophage-produced dual-tropic viruses are indeed preferentially interacting with DC-SIGN (or potentially other C-type lectins that can bind HIV-1 Env in a glycan dependent manner) over lymphocyte-derived variants. This may be one explanation for why HIV-1 in some individuals evolves from CCR5 usage to dual-tropism (61).

Our 2G12 neutralisation experiments confirm data from a previous study, which observed an 8- to 10-fold higher IC_50_ for macrophage-derived over lymphocyte-derived virus using chimpanzee serum (31). Differences in glycosylation modifications between various cell types may result in occlusion of part of the 2G12 binding site or directly affect the 2G12 epitope. Macrophages are known to produce viruses with an increased level of gp120 shedding (31), which may interfere with 2G12 neutralisation. However, it is unlikely that this explains the observed difference in neutralisation, since neutralisation correlates more with oligomeric than monomeric gp120 (62). Neutralisation experiments with H671-B10 will also provide insight into the relation between neutralisation with 2G12 and DC-SIGN binding and virus or Env antigen capture by DC-SIGN expressing cell types. Although the 2G12 epitope and the DC-SIGN binding site partially overlap (63), we observed equal DC-SIGN mediated transmission for macrophage- and lymphocyte-produced SF162, while macrophage-produced SF162 was more resistant to 2G12 inhibition. The 2G12 antibody has a more restricted epitope than DC-SIGN and binds to terminal mannose residues of specific potential N-linked glycosylation sites (PNGS) (53, 64). DC-SIGN preferentially binds internal trisaccharides and mutations of single residues do not affect the DC-SIGN binding site, while this can severely affect 2G12 binding (54, 63, 65, 66). Changes in glycosylation therefore more likely affect 2G12 neutralisation than DC-SIGN binding, which explains our findings on SF162. Since we observed differences in DC-SIGN-mediated transmission for H671-B10, we expect a more pronounced difference in sensitivity to 2G12 neutralisation than for SF162.

HIV-1 patients can be co-infected with pathogens such as *Plasmodium falciparum*, *Mycobacterium tuberculosi*s as well helminths and each induces a particular immune response. These differential immune responses in all likelihood influence HIV-1 replication by preferential infection of a particular cell type or virus inhibition by CC-chemokines. For example, a Th1 response impairs virus replication more than a Th2 response with increased CTL activity and decreased virus production by Th1 cells, compared to a Th2 milieu (1, 47, 67). Co-infection with *P. falciparum* increases virus production by macrophages (68). Further, we have previously observed modulation of DC-SIGN mediated HIV-1 trans-infection by *Mycobacterium tuberculous* (69) as well *Schistosoma mansoni* (70). Co-infecting pathogens may therefore influence virus replication and HIV-1 pathogenesis through stimulation of variant cell types within which HIV-1 replicates *in vivo*.

Our data indicate that virus production from different lymphocyte subsets, namely Th1 and Th2 cells, does not compromise infectivity for the alternate cell subset. This also does not result in significant differences in co-receptor affinity or virus capture via the DC-SIGN receptor as would be similar for viral gp120 antigen capture. When compared to lymphocytes, virus production by macrophages does not influence sensitivity to CC-chemokines but can affect DC-SIGN mediated transmission and sensitivity to 2G12 antibody neutralisation.

One limitation of this study is that Th1 and Th2 cells as well as macrophages were derived from PBMCs from a single donor, and so it is possible that the observations of this study are donor specific. Future investigations could aim to replicate these findings in cells derived from a wide range of donors. Despite this, these results give more insight to what extent the host cell influences viral phenotype and thereby various aspects of HIV-1 pathogenesis.

## Data availability statement

The raw data supporting the conclusions of this article will be made available by the authors, without undue reservation.

## Author contributions

EH, ED, GP and WP Devised the study. EH, TV performed the experiments. EH, JT, TV, ED, GP and WP analysed results. EH, JT and WP wrote the manuscript. All authors contributed to the article and approved the submitted version.
